# Data Collection Approaches to Enable Evaluation of a Massive Open Online Course About Data Science for Continuing Education in Health Care: Case Study

**DOI:** 10.2196/10982

**Published:** 2019-04-02

**Authors:** Abrar Alturkistani, Azeem Majeed, Josip Car, David Brindley, Glenn Wells, Edward Meinert

**Affiliations:** 1 Global Digital Health Unit Department of Primary Care and Public Health Imperial College London London United Kingdom; 2 Department of Primary Care and Public Health Imperial College London London United Kingdom; 3 Healthcare Translation Research Group Department of Paediatrics University of Oxford Oxford United Kingdom; 4 Oxford Academic Health Science Centre Oxford United Kingdom

**Keywords:** education, distance, education, teaching, online learning, online education, MOOC, massive open online course

## Abstract

**Background:**

This study presents learner perceptions of a pilot massive open online course (MOOC).

**Objective:**

The objective of this study was to explore data collection approaches to help inform future MOOC evaluations on the use of semistructured interviews and the Kirkpatrick evaluation model.

**Methods:**

A total of 191 learners joined 2 course runs of a limited trial of the MOOC. Moreover, 7 learners volunteered to be interviewed for the study. The study design drew on semistructured interviews of 2 learners transcribed and analyzed using Braun and Clark’s method for thematic coding. This limited participant set was used to identify how the Kirkpatrick evaluation model could be used to evaluate further implementations of the course at scale.

**Results:**

The study identified several themes that could be used for further analysis. The themes and subthemes include learner background (educational, professional, and topic significance), MOOC learning (learning achievement and MOOC application), and MOOC features (MOOC positives, MOOC negatives, and networking). There were insufficient data points to perform a Kirkpatrick evaluation.

**Conclusions:**

Semistructured interviews for MOOC evaluation can provide a valuable in-depth analysis of learners’ experience of the course. However, there must be sufficient data sources to complete a Kirkpatrick evaluation to provide for data triangulation. For example, data from precourse and postcourse surveys, quizzes, and test results could be used to improve the evaluation methodology.

## Introduction

### Background

Online learning in the form of massive open online courses (MOOCs) became internationally famous in 2011 when a Stanford University MOOC attracted learners from more than 190 different countries [1]. Although these courses have become heralded for their ability to attract a significant number of learners, their overall effectiveness is not well understood, especially considering most learners who start these courses do not finish them. MOOC evaluations can help analyze learning effectiveness and help improve their application [2]; however, there is a gap in the literature on MOOC evaluation methods [3]. Recent systematic reviews on MOOC research have concluded that there is a need for more research on methodologies used in MOOC research [4,5]. In addition, because of the diversity and heterogeneity of MOOCs, there is a need to focus on individual MOOCs and evaluate their effectiveness on a course level [6]. Current MOOC studies lack consideration of work-related skill development and organizational-level improvements [7]. A MOOC, especially one which focuses on practical skills development goals, should be assessed based on its quality of instruction, the inclusion of assessments, support of participation, instructional support, and enabling of continuous education [8]. Therefore, a MOOC evaluation should consider different aspects of the course instead of focusing on only limited aspects of learning.

Recent trends in MOOC research indicate there is an increase in using qualitative studies in MOOC research, which has been dominated by quantitative studies historically [7]. A quantitative approach tends to focus on course activity of the mass number of participants but without insight into individual activity. Qualitative methods and examination of individual learners provide contrasting data but are challenging to execute. Mixed-methods studies could enhance the methodological quality of this research by allowing for data triangulation from quantitative and qualitative data sources [4]. In addition, using more refined and sophisticated data collection and analysis methods such as interviews and focus groups and adopting thematic or social network analyses are highly recommended to improve MOOC evaluations [4]. There is a need for comprehensive and sophisticated data analyses methods to improve MOOC research.

### Objectives

Health iQ created a pilot MOOC called “Data Science Essentials: Real World Evidence” with the aim to introduce learners to the concept of real-world evidence and demonstrate the application of these methods across various health care and life sciences industries [9]. As the online course was a pilot run, it had a limited trial audience. The target audience of the course was described as “undergraduate students in data science, an analyst or commercial manager working in life sciences pharmaceuticals, healthcare regulation, biotech and medical devices, especially those with an interest in the application of Information and Communication Technologies (ICT) within healthcare” [9]. In this investigation, we sought to explore the success of the course’s objectives regarding “reach” about intended audience and social networks, “efficacy” about knowledge/skill gain skill and attrition, and adoption and sustainability of social networks for continual learning in this emerging field.

The objective of this study was to trial data collection methods to inform course development and to reflect on evaluation methodology for future course runs. Although an initial goal of the study was to perform an overall evaluation of the course using the Kirkpatrick evaluation method, because of time constraints and lack of data, we were only able to perform thematic analysis of the semistructured interview data. The purpose of the study was centered on the way semistructured interviewing could be used to implement the execution of a Kirkpatrick evaluation. The purpose for establishing an evaluation model that could be used in future MOOC evaluations is to be able to address research questions centered on the course’s impact on learners knowledge, skills and attitudes, and its effect on learners’ work and workplace.

## Methods

### Overview

This section will first provide an introduction about the course being studied and give an overview of the participants, data collection, and the data analysis methods used. This study employed semistructured interviews to analyze learner perspectives. The interview data were analyzed using thematic analysis methods. The Kirkpatrick evaluation model was used to organize and structure themes identified from interviews. We have reported the study methods and results according to the Consolidated Criteria for Reporting Qualitative Research (COREQ) [10]. The completed COREQ checklist can be found in Multimedia Appendix 1. The study received ethical approval from the Education Ethics Review Process (EERP) at Imperial College London (EERP1617-030).

### About the Course

Data Science Essentials: Real World Evidence was run twice, during August to September and October to November 2017. In total, 191 learners joined both runs of the course, where 56 were from the October cohort [11]. The course learning outcomes and facilitation have been described previously [11].

### Participants

All course participants were invited to be interviewed for the study via email through purposive sampling. A total of 7 learners had expressed interest to be interviewed, out of which only 2 chose to participate following informed consent [11]. Participants who dropped out did not provide any reasons. Interviewed participants’ gender was 1 male and 1 female. Participants’ age was not recorded, but only adults older than 18 years were able to participate in the study. Both participants were professionals working in health care–related fields, a medical doctor working in the pharmaceutical industry and a health care economist working in a consultancy organization.

### Data Collection

The interviews were conducted in December 2017 through conference calls [11]. Only the participant and interviewer were present in the interview [11]. An interview guide with the key topics and questions was used to help focus on the topics of interest. The guide included the interview questions and possible follow-up questions. Questions were centered on the participant’s background, reasons for taking the course, participant’s use of the learning in the workplace, participant’s interaction with other learners, and participant’s opinions about the different materials and tools used to deliver the course. Each interview lasted approximately 20 to 40 min and was audio recorded. Interview transcription was performed by the researcher as a way to start data familiarization [12]. The interviewees did not have any personal or professional relationship with anyone from the research team.

### Data Analysis

Data analysis was completed by performing thematic analysis. Interview recordings were transcribed verbatim, anonymized, and analyzed [13-15]. The semistructured interview questions were grouped into 3 sections: learners’ occupation and interests, learners’ application of the learning, and learners’ networking in the course. The participants were first asked about their background and their reasons for joining the course. The next questions were mainly focused on learners’ behavior after the course. For example, learners were asked whether they were able to apply learning in their work or studies and whether the course affected their data analysis skills. Participants were also asked about their engagement with other learners and their engagement with the course, and their feedback about these aspects was collected to collect data about networking in the course. The primary author conducted the interviews and (a female research assistant with training in qualitative research) was the primary data coder. Thematic analysis of the data was carried out using Braun and Clarke’s framework for thematic data analysis consisting of 6 phases: familiarization with data, generation of initial codes, searching for themes, reviewing themes, defining and naming themes, and production of a report [13-15]. Revision and verification of the codes were carried out through discussions with the principal investigator in each phase of the coding.

Data management before coding included removing interview questions from the transcripts to keep the coder focused on the primary purpose of the research. Preliminary coding occurred through the transcription of the interviews, reading and rereading of the data, and systematically open coding the data [13-15]. Coding was performed manually using Microsoft Word, and preliminary codes were organized in an Excel sheet to be reviewed by the principal investigator. We have used inductive coding, meaning that the themes formulated were data-driven [16].

Thematic analysis is one of the most used methods in qualitative studies, and interpreting data by forming themes is “the most applicable” method of analysis for interview data [16]. Previous evaluations of educational and training programs have used thematic analysis for the interpretation of data such as interviews, surveys, and discussion posts [17-19].

### Kirkpatrick Evaluation Model

The Kirkpatrick evaluation focuses on 4 levels of a training program: reaction, learning, behavior, and results [20]. This method could be used to evaluate participants’ opinion about the course (reaction); whether the participants learned from the course (learning); whether they experienced any consequent changes in behavior (behavior); and how this impacted their studies, work, or broader community (results) [18]. Kirkpatrick evaluation provides a practical and systematic method for evaluating a training program, and it was used previously in MOOC evaluations [21-23]. The semistructured interviews can address some of the Kirkpatrick model’s evaluation levels, but there is still a need for further data collection to fully validate the 4 levels of the model. In the following paragraphs, we describe the components of the model that could be covered using the semistructured interview data. Below we discuss the elements of the evaluation model that could be addressed by the semistructured interviews.

#### Level 1: Reaction

This level of the Kirkpatrick model evaluates participants’ overall reaction to the course and their opinions about the delivery of the course. Information such as why the learners joined the course, what they liked or disliked about the course, and how much they have completed of the course could be reported in this level of the model.

#### Level 2: Learning

This level of the model evaluates learning gained from the course. It can evaluate how well participants acquired new information or new skills through the course.

#### Level 3: Behavior

This level of the model should evaluate the behavioral change that participants were able to adopt as a result of taking the course. For example, this level could evaluate whether participants were able to create change in their workplace as a result of taking the course, whether this change (if any) was sustainable, and whether they were aware of a shift in their behavior.

#### Level 4: Results

This level of the model assesses whether differences were made for the participants’ workplace or organization as a result of the learning. This level of the model might be best evaluated after the course to allow time for the changes to occur.

## Results

The thematic analysis resulted in the following themes: learner background, MOOC learning, and MOOC features [11].

### Thematic Analysis Results

**Figure 1:**
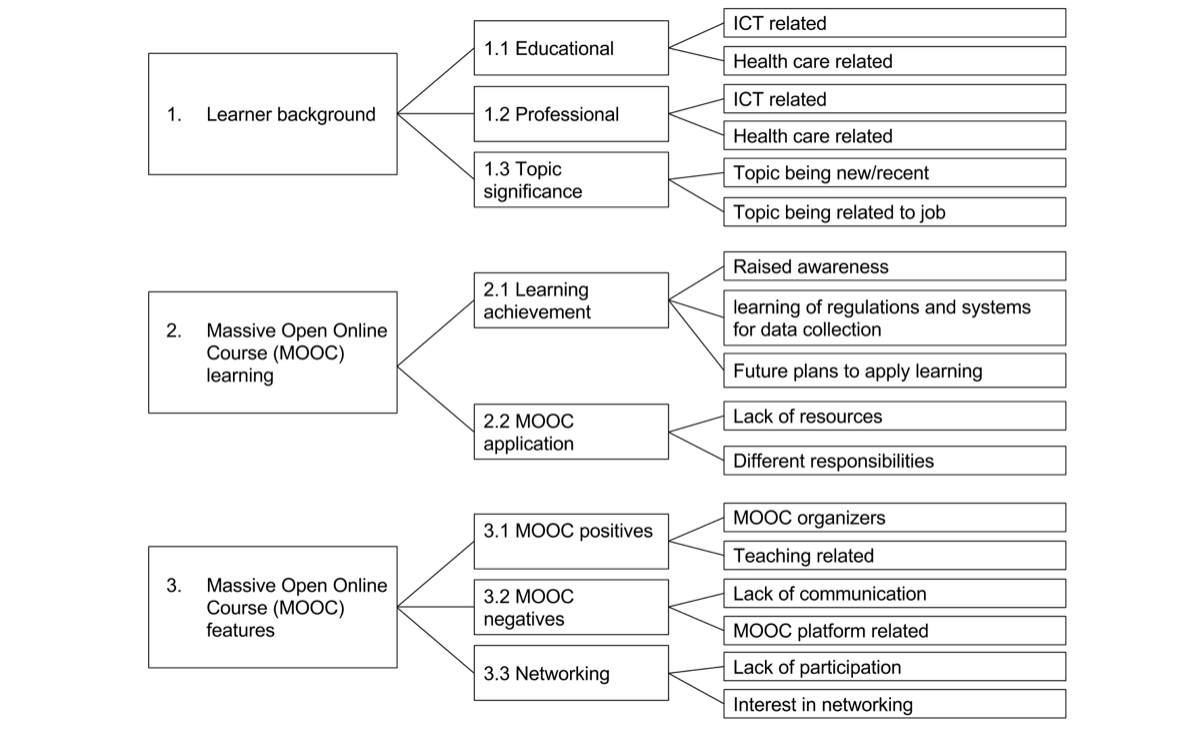
Themes, subthemes and codes developed through thematic analysis of interview data. ICT: information and communication technologies.

Analysis of the semistructured interview data gave rise to 3 central themes: learner background, MOOC learning, and MOOC features. Each of the themes and their subsequent codes from the thematic analysis of semistructured interview data (adapted from the study by Alturkistani et al [11]) are shown in Table 1. Figure 1 shows the themes, subthemes and codes developed through thematic analysis of interview data. Complete results of the thematic analysis can be found in Multimedia Appendix 2. The results were based on the 2 learners’ responses. For that reason, it cannot be said that data saturation was reached; therefore, the study outcomes were limited to the view of the 2 learners only.

**Table 1:** Themes, subthemes, and codes from the thematic analysis of semistructured interview data.

Themes and subthemes		Codes
**Learner background theme**		
	Educational	Information and communication technologies–related and health care–related
	Professional	Information and communication technologies–related and health care–related
	Topic significance	Topic being new/recent and topic being related to job
**MOOC^a^** **learning theme**		
	Learning achievement	Raised awareness, learning of regulations and systems for data collection, and future plans to apply learning
	MOOC application	Lack of resources and different responsibilities
**MOOC features theme**		
	MOOC positive	MOOC organizers and teaching-related
	MOOC negatives	Lack of communication and MOOC platform–related
	Networking	Lack of participation and interest in networking

^a^MOOC: massive open online course.

#### Theme 1: Learner Background

Learners’ educational background included undergraduate clinical medical training, a Masters in Economic Evaluation in Health Care, and Masters in Biostatistics, and their professional experience included working in the pharmaceutical industry and the health care sector and being involved with data science at work. The codes ICT–related and health care–related represent learners’ educational and professional fields that were closely related to the course’s field of interest, the intersection of ICT with health care.

#### Theme 2: Massive Open Online Course Learning

Participants expressed their learning through different methods such as expressing the different topics that they have learned through the course. They have also discussed how they were able or not able to apply learning in their work or studies.

#### Theme 3: Massive Open Online Course Features

Each participant had different opinions about what they liked and disliked about the MOOC and their experience in networking.

### Reflection on the Kirkpatrick Evaluation Model

#### Level 1: Reaction

The reaction level of the model could be collected through the semistructured interviews. The participants’ reaction to the course could easily be collected through the semistructured interview. Completion rates of learners could be collected through the interview but can also be recorded through the learning management system data, which can automatically report the completion rate of the different components of the course.

#### Level 2: Learning

Overall, it is possible to ask participants how much and how well they have learned in the course through the semistructured interviews. However, it may be useful to collect data through quiz or test scores, if possible, to triangulate and strengthen the interview findings.

#### Level 3: Behavior

It is possible to ask participants about the different behaviors they have changed as a result of taking a course. However, behavior change is one of the least studied outcomes in MOOC research, and it may be challenging to only record it through the semistructured interviews. When learners were asked if they have engaged in different projects as a result of taking the course, they have responded negatively. It may be useful to enhance the results of this level of the evaluation by collecting data through postcourse surveys possibly in 2 different time points, right after the course and 3 to 6 months after the course to allow some time for changes after the MOOC.

#### Level 4: Results

On the basis of the course description, the aim of the course was to teach learners how to “develop new methods for data analysis” and use of the data to “inform decision making in health care” [9]. Therefore, the potential impact of course would have been to demonstrate that new methods of data analysis were adopted and that the new data informed decisions in health care. The data for this level of the evaluation could be collected both through semistructured interviews and postcourse surveys.

## Discussion

### Principal Findings

This study gathered data to consider the use of semistructured interviews to inform a proposed evaluation method. Thematic analysis of semistructured interview data with learners of the pilot run of the course was completed to identify key themes for future development of the course. The Kirkpatrick evaluation model components were reviewed to assess whether semistructured interview data could help evaluate the course. The trial interview process revealed that the Kirkpatrick evaluation model could be used through the semistructured interview data in addition to other data sources such as surveys and quizzes. Semistructured interviews, while providing in-depth data about the learners’ experience, may be a limited method to record objective data on things such as learning, behavior, and results.

A review of the recent MOOC literature (2013-2017) found that there is limited literature on studies focusing on learners’ acquired practical skills from MOOCs [8]. In general, MOOC evaluations have not yet been able to measure the long-term impacts of MOOCs on learners [24]. However, the use of methods to measure course impact, including the Kirkpatrick evaluation model with its consideration of behavior change and results on the organizational level can help take learner skills and behavior change into account when evaluating the course. In a subsequent study, use of this method could be conducted by collecting pre- and postcourse surveys, quiz, and test results and possible discussion posts and triangulating this information with semistructured interviews data.

Our study’s strengths are that it used qualitative data to assess the applicability of evaluating learning and skills of participants after the course. A recent systematic review (2018) of MOOC research recommended that methods such as interviews that offer an in-depth data of learner or participant experiences should be preferred to survey and “easily obtainable descriptive statistics” data [4]. It is believed that studying the success of an online learning course should focus more on the applicability of the information to the learners’ day-to-day activities [25]. Our study suggests that evaluations should focus on how learning can affect that participant’s behavior and work.

The limited qualitative data we collected informed us what factors need to be examined in more depth to evaluate the effectiveness of a MOOC and could help researchers consider factors beyond learners’ knowledge to understand what can help improve the MOOC’s applicability in real life. Future evaluations could include more data sources such as surveys, discussion posts, and quiz results when using the Kirkpatrick model [21] to increase the reliability of analysis. Furthermore, studies could use learning analytics data that are recorded through the host online course website of learners’ use of the course (eg, login details and video viewing activity) to have a more comprehensive understanding of MOOC activity [26]. The main limitation of the study was the small sample size, which limits the generalizability of our study. The small sample size also meant that we were not able to fully address the study research questions. Due to the lack of data, we were unable to use any precourse measurements to compare participants’ reaction before and after the course or report demographic information about the target population of the course. We also relied entirely on participants’ self-reported data, which are subject to bias. However, this was a pilot study to inform our future course evaluations, and the limitations were taken into account when reporting the outcomes of the study.

### Conclusions

The core themes that resulted from this study indicate that MOOCs could potentially be evaluated in terms of their impact on learners’ behavior and skills acquired from the course through performing the Kirkpatrick evaluation. The study concluded that semistructured interviews can provide valuable, in-depth data about the course but should be used along with other data sources for data triangulation. Data sources such as pre- and postcourse survey data, quiz and test scores data, and possible discussion or social media thread posts could help create a comprehensive evaluation using the Kirkpatrick evaluation method.

## Appendix

Multimedia Appendix 1 Consolidated Criteria for Reporting Qualitative Research (COREQ) checklist.

Multimedia Appendix 2 Thematic analysis results.

## Abbreviations

COREQ: Consolidated Criteria for Reporting Qualitative Research
EERP: Education Ethics Review Process
HEFCE: Higher Education Funding Council
ICT: information and communication technologies
MOOC: massive open online course
