# Supplementary material for: Data Collection Approaches to Enable Evaluation of a Massive Open Online Course About Data Science for Continuing Education in Health Care: Case Study
Source: JMIR Med Educ. 2019 Apr 2;5(1):e10982. doi: 10.2196/10982 (PMC6465971; doi:10.2196/10982)
Supplement: Multimedia Appendix 1 [file mededu_v5i1e10982_app1.pdf]

Multimedia Appendix 1: Consolidated criteria for reporting qualitative research (COREQ) checklist

Checklist developed from:
Tong A, Sainsbury P, Craig J. Consolidated criteria for reporting qualitative research (COREQ): a 32-item checklist for interviews and focus groups. Int J Qual Health Care 2007 Dec;19(6):349–357. PMID:17872937

No. Item	Guide questions/description	Reported on Page #
**Domain 1: Research team and reflexivity**		
Personal Characteristics		
1. Interviewer/facilitator	Which author/s conducted the interview or focus group?	4,11
2. Credentials	What were the researcher's credentials? E.g. PhD, MD	11
3. Occupation	What was their occupation at the time of the study?	4
4. Gender	Was the researcher male or female?	4
5. Experience and training	What experience or training did the researcher have?	4
**Relationship with participants**		
6. Relationship established	Was a relationship established prior to study commencement?	4
7. Participant knowledge of	What did the participants know about the	4

the interviewer	researcher? e.g. personal goals, reasons for doing the research	
8. Interviewer characteristics	What characteristics were reported about the inter viewer/facilitator? e.g. Bias, assumptions, reasons and interests in the research topic	4
**Domain 2: study design**		
Theoretical framework		
9. Methodological orientation and Theory	What methodological orientation was stated to underpin the study? e.g. grounded theory, discourse analysis, ethnography, phenomenology, content analysis	4-5
Participant selection		
10. Sampling	How were participants selected? e.g. purposive, convenience, consecutive, snowball	4
11. Method of approach	How were participants approached? e.g. faceto-face, telephone, mail, email	4
12. Sample size	How many participants were in the study?	4
13. Non-participation	How many people refused to participate or dropped out? Reasons?	4
Setting		
14. Setting of data collection	Where was the data collected? e.g. home, clinic, workplace	4
15. Presence of nonparticipants	Was anyone else present besides the participants and researchers?	4

16. Description of sample	What are the important characteristics of the sample? e.g. demographic data, date	4
Data collection		
17. Interview guide	Were questions, prompts, guides provided by the authors? Was it pilot tested?	4
18. Repeat interviews	Were repeat interviews carried out? If yes, how many?	na
20. Field notes	Were field notes made during and/or after the interview or focus group?	4
21. Duration	What was the duration of the interviews or focus group?	4
22. Data saturation	Was data saturation discussed?	9
23. Transcripts returned	Were transcripts returned to participants for comment and/or correction?	na
**Domain 3: analysis and findings**		
Data analysis		
24. Number of data coders	How many data coders coded the data?	4
25. Description of the coding tree	Did authors provide a description of the coding tree?	na
26. Derivation of themes	Were themes identified in advance or derived from the data?	5
27. Software	What software, if applicable, was used to manage the data?	5

28. Participant checking	Did participants provide feedback on the findings?	na
Reporting		
29. Quotations presented	Were participant quotations presented to illustrate the themes/findings? Was each quotation identified? e.g. participant number	Multimedia Appendix 2
30. Data and findings consistent	Was there consistency between the data presented and the findings?	7-8
31. Clarity of major themes	Were major themes clearly presented in the findings?	6-7
32. Clarity of minor themes	Is there a description of diverse cases or discussion of minor themes?	7-8
