# Supplementary material for: Data Collection Approaches to Enable Evaluation of a Massive Open Online Course About Data Science for Continuing Education in Health Care: Case Study
Source: JMIR Med Educ. 2019 Apr 2;5(1):e10982. doi: 10.2196/10982 (PMC6465971; doi:10.2196/10982)
Supplement: Multimedia Appendix 2 [file mededu_v5i1e10982_app2.pdf]

Multimedia Appendix 2: Thematic analysis results:

Thematic analysis results have been reported briefly in: Alturkistani A, Car J, Majeed A, Brindley D, Wells G, Meinert E. Determining the effectiveness of a massive open online course in data science for health. Madrid, Spain; 2018. ISBN: 978-989-8533-78-4

Analysis of the semi-structured interview data gave rise to three central themes: 'learner background', 'MOOC learning' and 'MOOC' features.

Theme 1: Learner background.

Sub-theme 1.3: Topic Significance.
Topic being new/recent
Participants were especially interested in real world evidence, and the fact that the topic being first of its kind:
"I wanted to get a better understanding of...what defines real world evidence in the marketplace because it sounds like a Buzzword, I just wanted to know like, for validation really" (p1)

Topic being related to job
Participants joined the MOOC because the topic was relevant to their job:

"The reason I joined this course is because I anticipated that having, being equipped with this knowledge put me in a better position within my job" (p1)

"I am currently working as a medical science liaison in pharmaceutical industry...currently my role, I am also responsible for Real World data in my company in the [eliminated word] region and its like taking, its only 20% of my time in the job description" (p2)

Theme 2: MOOC learning.
Sub-theme 2.1: Raised awareness.

"the part of the information governance was very new for me, ... just it puts the whole process into perspective, in a system, like now I know that there is a system existing for payroll data and pharmaceutical, academic collaboration, and they were many excellent case studies" (p2)

Learning of regulations and systems for data collection

Both participants expressed their learning from the MOOC by talking about some of the topics they have learned about like “governance”, “regulations” and “systems” related to data collection.

Future plans to apply learning
Participants were confident about using the information in the future. For instance, one participant expressed that even though their workplace was not yet ready to implement what was learned from the MOOC, the information learned will be very likely to be used in the future:
“I'm sure I will get back to them one day.” (p2). Also, the same participant has added about the regulations taught in the MOOC: “...I believe, they will give, they are a very good example of the existing regulations, and also different resources and sources of data sets, I believe this will be very helpful.” (p2)

Also, the other participant has described the MOOC as being a “good course,” that it was “taught better” than a different MOOC about a similar topic and explained that these qualities of the MOOC ensure the use of learning in the future.

Sub-theme 2.2: MOOC application.
Participants had similar reactions to the application of the MOOC in their work or studies; both were not able to apply the learning from the MOOC. The barriers to the use of the learning from the MOOC were reflected in the following codes:

Lack of resources
Lack of resources expressed as a lack of data sources:
"currently in my current role, we, unfortunately, don't have a data source, but we are planning to discover and to develop some, but I should be, I am assigned to do this, but we did not start yet” (p2)

Lack of resources expressed as lack of time
“so that is difficult to say because I was like in the final two weeks of my job and that health economics consultant, when I started this MOOC, so I didn’t really get to do the applying my knowledge in my work,” (p1)

Different responsibilities
“I probably would say that I did not use it yet because the projects that I work, well the clients that we work with um, they didn't, they're not at the stage of, well they didn't come to us for that part of data collection ..." (p1)

Theme 3: MOOC features.

Subtheme 3.1: MOOC positives.
Each participant had different opinions about what they liked most about the MOOC.

MOOC organizers
“and I just, I was happy to find out that there is a course, an online course about it, by the Imperial College.” (p2)

Teaching related
One participant found the videos to be the most engaging as a teaching method:
“The videos were the most engaging. I like both. I like the videos and the articles, but the videos were more engaging for me. they are easier to follow maybe” (p2)

The other participant found the assessments and the up-to-date recommended links to be the most helpful:
"I like the questions throughout the lesson because it does test you, whether you're actually concentrating or flicking through the MOOC." (p1)

“I did really like the links at the end, where you could find more information, because they were pretty up to date, in the past, in very recent years,” (p1)

Sub-theme 3.2: MOOC negatives.
Lack of communication between learners
Participants have expressed not being able to communicate with other learners negatively. One participant used the words “unfortunately not,” (p2) when asked if they were able to communicate with other learners, while the other expressed the experience:
“I lacked the motivation, not knowing who else was doing it...it did feel like a very, very independent experience...Yes, because, well I couldn’t tell who else was learning on the course,” (p1)

MOOC platform related
The other negatives mentioned about the MOOC were more related to the technical issues with the MOOC platform itself. One participant did not find the platform to be “user-friendly” and both believed it would be better if there were more features available on the platform that could allow things like pausing lessons and returning to the same place after logging off and being able to download the videos for offline viewing.

Sub-theme 3.3: Networking.
Participants did not communicate or network with others during or after the MOOC, and the reasons seemed to overlap.

Lack of participation

Initially, participants were interested in networking or communicating with other learners who were evident in their attempt to visit the social media page created for the MOOC (accessible through a Twitter hashtag). The inactivity and lack of participation in the platform was the main reason for not networking.

“ when I first started the course, I think it was like the first two weeks, so I looked at the hashtag, which encourage conversation on Twitter, but I did notice that there wasn’t that much going on, I guess because there were so few people actually speaking or having a conversation about those topics, that I ended up not going forward with joining in the conversation...” (p1)

"Unfortunately not, I tried to follow at the beginning the hashtag of the course on Twitter, but I didn't find it very active, so I didn't follow up after the first week...So I didn't initiate any conversations." (p2)

Interest in networking

Lack of networking during this MOOC did not mean that participants were not interested in networking as they have expressed interest in it.

“it is a very good idea actually. It would be a very good opportunity because you would share relevant material and relevant news and opportunities, yes” (p2)
